# Impact of the COVID-19 pandemic on the codification of China’s civil code

**DOI:** 10.3389/fpubh.2024.1415578

**Published:** 2024-10-17

**Authors:** Gaoyuan Zhai, Xiaochang Liu

**Affiliations:** Law School, Tianjin University, Tianjin, China

**Keywords:** China, civil code, COVID-19, COVID-19 provisions, epidemic prevention and control

## Abstract

The COVID-19 pandemic has spread widely around the world since 2019, wreaking havoc on social order in a global context. In China, the pandemic has not only provided new challenges to the government’s capacity but also provided new issues for the Civil Code that is being compiled. The study adopts the research method of comparative analysis and combines the case study method, aiming to study the impact of the epidemic on the compilation of China’s Civil Code. We found that the official Civil Code adds, deletes, and adjusts several articles compared to the Civil Code (Draft), five of which are relevant to the COVID-19 pandemic. The Civil Code was enacted at the time of the COVID-19 pandemic, which led to some of its provisions being affected by the COVID-19; the “COVID-19 provisions” not only provided legitimacy for the prevention and control of the pandemic but also improved China’s system of governance of public health emergencies. This study exposes the legal loophole in the “COVID-19 provisions” in China’s Civil Code and further suggests ways to fill the legal loophole.

## 1 Introduction

On 1 December 2019, China’s first patient of COVID-19 infection was diagnosed in Wuhan (1). COVID-19 rapidly spread from Wuhan to other provinces and regions, and the number of diagnosed patients started to increase on a daily basis. By 24:00 on 7 December 2022, the Chinese mainland had reported a cumulative total of 354,017 confirmed patients and a cumulative total of 5,235 deaths (2). The COVID-19 pandemic broke out in the final stages of the codification of the Chinese Civil Code, and the sudden public health event affected the promulgation of the Civil Code. Until 28 May 2020, China’s 13th National People’s Congress voted to adopt the Civil Code (3) in its third session. Compared to the Civil Code (Draft) published on 16 December 2019, the official Civil Code adjusted several provisions, five of which had a close relationship with the COVID-19 pandemic.

These provisions include the following: Article 34, paragraph 4, which created the temporary guardianship system; Article 245, which added “prevention and control of epidemics” to the reasons for requisition; Article 285, paragraph 2, which stipulated the responsibility of property management service enterprises to carry out emergency response measures; Article 286, paragraph 1, which stipulated the owner’s obligation to cooperate with the property management service enterprise; Article 494, paragraph 1, added “prevention and control of epidemics” to the reasons for initiating State compulsory contracting.

As the above five provisions are related to the COVID-19 epidemic prevention and control, they are called the “COVID-19 provisions” of the Civil Code.

## 2 The civil code created the temporary guardianship system

Compared with the official Civil Code, Article 34 of the 16 December 2019 version of the Civil Code (Draft) had only three articles and no provision for temporary guardianship. During the COVID-19 pandemic, there were many incidents in China where minors or persons with disabilities lacked guardianship, and these incidents prompted the creation of temporary guardianship provisions in the Civil Code.

### 2.1 The COVID-19 pandemic and lack of guardianship

At the early stage of the COVID-19 pandemic, the Chinese government required all people to be quarantined at home, and medical institutions quarantined people who had been found to have symptoms of COVID-19 to prevent the spread of the pandemic. In the process of epidemic prevention and control, the inflexible quarantine measures caused a series of tragedies, the most famous of which was the “Y incident.” Y was a child from Huanggang City, Hubei Province, who suffered from cerebral palsy and severe intellectual disability and could not take care of himself. On 23 January 2020, Y’s father was forced to quarantine because he was suspected of being infected with COVID-19. Y stayed in isolation at home alone and eventually died on the afternoon of January 29. Y incident aroused widespread discussion, with people questioning and criticizing the ability of the local grassroots government. During the period of quarantine, Y’s father could only seek help online, and finally, the Association for the Disabled and Social Welfare Organizations requested the villagers’ committee to provide basic necessities for Y. During the 6 days that Y was isolated alone, the villagers’ committee claimed that Y ate something on the 24th and 26th and drank two cups of amino acids on the 28th (4). The care and food provided to Y by the village committee were clearly unable to support his basic living, and the village committee’s negligence facilitated the process of Y’s death. The Y incident profoundly reminded us how to safeguard the life and health of wards who lose guardians during public health emergencies.

In fact, the Y incident was not an isolated case during the COVID-19 pandemic. There were also cases such as “6-year-old child left alone at home for days after grandfather died” (5) and “6-month-old baby left alone in hospital quarantine after all family members had been diagnosed” (6). It can be seen that during the pandemic, many vulnerable people struggle to survive because of separation from their guardians, and their lives and health are fraught with uncertainties under the risk of the pandemic.

### 2.2 Inadequacies in the temporary guardianship provision

To fill the legal loophole of lack of guardianship in emergency situations, the legislature added paragraph 4 to Article 34 of the Civil Code, “Where a guardian is temporarily unable to perform his duties owing to an emergency such as an unexpected incident, thus leaving the ward in an unattended situation, the residents’ committee, the villagers’ committee, or the civil affairs department in the place where the ward’s domicile is located shall make arrangement as a temporary measure to provide necessary life care for the ward.” This provision safeguards the human rights of vulnerable groups and reflects the people-oriented legislative philosophy of the Civil Code (7).

However, the practice of having residents’ committees, villagers’ committees, or the civil affairs department arrange the necessary and temporary living measures for the wards is flawed and prone to guardianship failures (8). During emergencies, the departments not only have to deal with many problems caused by emergencies but also have to take on additional guardianship responsibilities, and the high workload is not conducive to the quality of guardianship for the wards. This study holds that relatives are better suited to serve as temporary guardians than the departments provided for in the Civil Code, and it is family and blood-based kinship that may break through secular concepts to truly play a role in safeguarding the interests of wards. Relatives help each other out of ethical obligation and kinship (9). In the Y incident, while Y was isolated alone, medical staff refused to come into close contact with Y because they lacked medical protective clothing, but Y’s aunt took care of him many times despite the risk of infection.

Biologist William Donald Hamilton argued that altruistic behavior generally occurs between kin and is proportional to the degree of consanguinity; the closer the individuals are to each other, the stronger the tendency to be altruistic toward each other (10). Relatives acting as temporary guardians avoid the guardianship drawbacks of government departments guarding more than one person at the same time and can relieve the work pressure of government departments and utilize their limited resources for emergencies. The structure of human relationships in China is influenced by Confucianism, and the quality of guardianship is ensured by the kinship between the guardian and the ward. Therefore, this study holds that it is more in the best interests of the ward for a relative to act as a temporary guardian, instead of a social worker or government staff.

The logical consistency of the legal system requires that the constituent elements of a civil offense should correspond to civil liability (11). Article 34, paragraph 4, of the Civil Code stipulates that the residents’ committee, the villagers’ committee, and the civil affairs department shall guarantee the basic livelihood of the ward. What are the responsibilities of these organizations if they fail to fulfill their responsibilities? Article 179 of the Civil Code stipulates that the main forms of civil liability include the following: (a) cessation of the infringement; (b) removal of the nuisance; (c) elimination of the danger; (d) restitution; (e) restoration; (f) repair, redoing, or replacement; (g) continuance of performance; (h) compensation for losses; (i) payment of liquidated damages; (j) elimination of adverse effects and rehabilitation of reputation; (k) extension of apologies. We can find that none of the above 11 types of civil liability are appropriate for breaches of the duty of temporary care. The provision of temporary guardianship in the Civil Code solves one problem but also brings about another new one.

This study argues that legal responsibility for breaches of the duty of temporary care should be administrative. Article 34, paragraph 4, of the Civil Code is not a civil law provision but an administrative law provision (12). Purely administrative legal norms appear in civil legal norms, which ultimately lead to confusion in the legal system. This study argues that since it is a purely administrative legal norm, paragraph 4 should be deleted from Article 34 of the Civil Code and thus regulated in the corresponding administrative legal normative document.

## 3 The civil code added “prevention and control of epidemics” to the reasons for requisition

During the COVID-19 pandemic, Wuhan was under lockdown from 23 January to 7 April 2020, and by 24:00 on 7 April 2020, Wuhan had had a total of 5,008 confirmed patients and a large number of close contacts (13). The large number of confirmed patients, as well as close contacts, has led to a severe shortage of medical resources in Wuhan and enormous pressure on medical institutions. To provide timely treatment for patients infected with COVID-19 and to isolate close contacts, Wuhan has requisitioned private hospitals, hotels, gymnasiums, and university dormitories as centralized treatment and isolation facilities. During the epidemic, the Chinese government requisitioned social resources to relieve the huge pressure of epidemic prevention and control.

### 3.1 The COVID-19 pandemic and improper requisition

On 2 February 2020, the Health Bureau of Dali City, Yunnan Province, issued an announcement stating that due to the extreme shortage of epidemic prevention items in Dali City, nine boxes of masks shipped from Ruili City, Yunnan Province, to Chongqing City were requisitioned (14). This incident triggered a crisis of public opinion in the local government. Yunnan Province claimed that the actions of the Dali City Health Bureau had seriously hurt the feelings of the people of Yunnan Province and Chongqing City (15). In fact, the actions of the Dali City Health Bureau are illegal. According to Article 45 of China’s “Law on the Prevention and Control of Infectious Diseases,” the Dali Municipal Government can only requisition materials within its own administrative region. However, the Dali City Health Bureau violated the regulations within its own administrative region and requisitioned medical supplies sent from Ruili City to Chongqing City. This behavior violated the mandatory provisions of the law and was an act beyond its authority. Not only that, Article 11 of China’s Legislation Law stipulates that the objects of expropriation cannot be state-owned properties, and the masks were purchased by the Chongqing Municipal Government and are state-owned assets. Therefore, the behavior of the Dali Health Bureau also violated the mandatory provisions of the Legislative Law (16).

In addition to this, during the COVID-19 pandemic, the Wuhan government requisitioned many university dormitories to quarantine close contacts. Due to the unreasonable implementation of expropriation measures, students’ private property was damaged, triggering dissatisfaction and protests among students (17). The property lawfully owned by a private individual is protected by law, and no organization or individual may misappropriate, loot, or destruct such property. Even in emergencies, expropriation procedures should be legal and should take into account the interests of the above-mentioned organizations or individuals or at least minimize their losses.

### 3.2 The reason for “preventing and controlling epidemics” is unnecessary

The legislature revised the Civil Code’s requisition provisions based on experiences and lessons learned during the pandemic. Article 245 of the Civil Code provides that “an immovable or movable property of an organization or individual may, in response to an emergency such as providing disaster relief and preventing and controlling pandemics, be requisitioned within the scope of authority and pursuant to the procedures provided by law. The requisitioned immovable or movable property shall be returned to the aforementioned organization or individual after use. Where the immovable or movable property of an organization or individual is requisitioned, or where it is destructed, damaged, or lost after being requisitioned, compensation shall be made.” Compared to the Civil Code (Draft) published on 16 December 2019, the article adds “preventing and controlling epidemics” to the reasons for requisition, providing a legal basis for initiating expropriation during the epidemic.

However, this study holds that the reasons for requisition in the Civil Code (Draft) actually include “epidemic prevention and control,” so there is no need to add this reason for requisition. The main reasons include the following: first, the Civil Code (Draft) stipulates that the reason for expropriation is an emergency response and disaster relief, and pandemics endanger human health and destabilize societies, so the prevention and control of epidemics also belongs to the content of disaster relief; second, Article 245 of the Civil Code (Draft) is an open-ended provision that includes other reasons for requisitioning of the same type as emergency response and disaster relief, and epidemic prevention and control is one of the cases that is not enumerated in this category; third, if there are other reasons for a requisition that are not covered by Article 245 of the Civil Code in the future, such as the “state of war,” should the Civil Code add them to that article as well? If the legislature continues to enumerate the reasons for requisition, the article will become bloated and complicated.

Legal provisions should be concise, rigorous, and logical (18). In light of the foregoing, this study concludes that there is no necessity for Article 245 of the Civil Code to add “epidemic prevention and control” to the list of reasons for requisition.

## 4 The civil code stipulated the responsibility of property management service enterprises to carry out emergency response measures

The community is an important place in preventing the spread of the epidemic. With a large population in the community, any unreasonable management measures may lead to large-scale infection. It is a huge challenge for property management service enterprises to protect the basic livelihoods of owners while preventing and controlling the epidemic.

### 4.1 The COVID-19 pandemic and community management

During the pandemic, some property management service enterprises effectively safeguarded the lives, health, and property rights of owners, prevented the spread of the epidemic, and made important contributions to epidemic prevention and control. For example, in the early stage of the COVID-19 pandemic, the property management service enterprise of Wuhan Greenland Hankou Center, strictly controlled access to the gated residential community, sprayed disinfectant drugs several times, and a series of measures prevented the epidemic from spreading. As of 19 February 2020, there were still no people infected with the new coronavirus in the community, even though large-scale infections had already taken place in Wuhan. Not only that, this management model preceded the Wuhan government’s decision by more than 20 days, successfully shaping the barrier of life and health protection for the owners.

While property management service enterprises can play an important role in a pandemic, they are often challenged by the owners because of the lack of coercive power (19). Article 2 of China’s Property Management Regulations stipulates that property management enterprises are corporate bodies and do not belong to the state authorities, so they have no power to take relevant measures to restrict the freedom of owners. The management model of property management enterprise is mainly actualized through contractual means under the guidance of the government, and the cooperation is very fragile (20). In particular during the pandemic, the unstable living environment exacerbated people’s dissatisfaction, and due to the lack of coercive power in the management of property management service enterprise, flexible management is easily challenged by the owners. For example, a retired official in Jilin Province forcibly led other vehicles into the community (21), and a man without passes drove into the community by force (22), as often happened during the pandemic.

### 4.2 Preventing the expansion of the power of community managers

To resolve conflicts between property management service enterprises and the owners, the legislature empowered property management service enterprises through the Civil Code. Article 285, paragraph 2, provides that “the property management service enterprise or other managers shall carry out emergency measures and other management measures implemented by the government in accordance with law and actively cooperate in the performance of the related service.” The paragraph provides a legal basis for the property management enterprise or other managers to implement emergency response measures in special circumstances such as epidemic prevention and control. In addition, Article 286, paragraph 1, of the Civil Code stipulates that with respect to the emergency measures and other management measures implemented by the government in accordance with law that “are carried out by the property management service enterprise or other managers, the owners shall, in accordance with law, be cooperative.” This provision clearly stipulates the owner’s obligation to cooperate with the property management service enterprise.

However, the expansion of power tends to breed corruption, and the property management service enterprises, as the direct management organizations of the community, have the chance to gain private benefits in the name of emergency management at the cost of the interests of owners. During the pandemic, people had to be quarantined at home to prevent the further spread of the virus, and living necessities were purchased and delivered by the property management service enterprises, and some of them intentionally raised their prices drastically and sold low-quality commodities, taking advantage of the opportunity to make profits from the epidemic prevention and control (23). These inappropriate behaviors intensify the conflict between the owners and the property management service enterprises. On 5 March 2020, when senior officials of the State Council inspected a community in Wuhan to check on the prevention and control of the epidemic, the property management service enterprise of the community deceived the senior officials by asking volunteers to pretend to provide the owners with supplies, which caused serious dissatisfaction among owners, many of whom protested against the fraud to the senior officials by shouting slogans such as “scam,” “all scams,” and “formalists” (24).

This study holds that the Civil Code grants power to the property management service enterprises, but the boundaries of that power should be clarified, so as to avoid situations in which owners’ interests are jeopardized by the expansion of their power.

## 5 The civil code improved the state ordering contract system

### 5.1 The COVID-19 pandemic and shortage of medical supplies

During the COVID-19 pandemic, the epidemic spread rapidly from Wuhan to the whole of China, and the number of diagnosed patients continued to increase, so that medical supplies such as masks, goggles, and medicines quickly became hot commodities in short supply. The early stages of the pandemic coincided with the Chinese Lunar New Year, when most factories had been shut down and were unable to produce enough medical supplies in time to fill the huge market gap, resulting in serious shortages of medical supplies in several cities. In Wuhan, due to the persistent shortage of medical supplies, confirmed patients were unable to receive timely treatment, and several hospitals had to solicit donations of medical supplies from the public. The shortage of medical supplies not only reduced the efficiency of epidemic prevention and control but also intensified people’s panic.

### 5.2 Improvement of the state order contract system

To solve the problem of shortage of medical supplies during the epidemic, the legislature perfected the provisions of the State ordering contract of the Civil Code. Article 494, paragraph 1, of the Civil Code stipulates that “where the State issues a State purchase order or a mandatory assignment in accordance with the needs such as emergency and disaster relief, pandemic prevention and control, or the like, the persons of the civil law concerned shall conclude a contract in accordance with the rights and obligations provided by the relevant laws and administrative regulations.” This provision provides legitimacy to the State’s compulsory contracting, and in times of public health emergencies, the State may place orders with medical material enterprises, which in principle may not refuse to sign contracts (25).

In the Civil Code (Draft) of 16 December 2019, Article 494 only stipulates that the State may arrange State ordering contracts on the basis of need, without specifying the reasons. Article 494 of the official Civil Code further clarifies the reasons for the conclusion of a State order contract based on the provisions of the draft and specifies what is meant by “in accordance with the needs,” which is “in accordance with the needs of disaster relief, epidemic prevention and control, or other needs.” The changes to Article 494 of the Civil Code were clearly influenced by the pandemic, and the lessons learned from epidemic prevention and control were used to promote the improvement of the State ordering contract paragraph, which provides legitimacy for the State to issue State purchase orders in the event of a public health emergency.

The Civil Code (Draft) uses a generalized legislative model for the reasons for State ordering contracts, with vague provisions that give the State freedom to interpret the paragraph (26), and the legislative model facilitates the entering into State ordering contracts. However, the generalized legislative model is highly uncertain, and the expansion of governmental power can easily lead to arbitrariness in decisions if there is no restriction on the scope of the applicable conditions (27). Article 494 of the Chinese Civil Code limits the scope of the reasons for State ordering contracts by means of an enumeration, which is not exhaustive due to the limitation of the length of the legal provisions and other reasons. This shift from completely generalized legislation to an eclectic legislative model that combines both generalization and enumeration reflects the advancement of legislative techniques in China’s Civil Code, which overcomes the rigidity of enumeration and the uncertainty of generalization while maintaining the flexibility of the law.

## 6 Conclusion

Ulrich Beck referred to the societies of the 20th century onward as risk societies, where human beings are constantly exposed to socially created risks that threaten their existence (28). The worldwide spread of COVID-19 has caused serious disruptions to the global social order (29); during the novel coronavirus pandemic, the global community was severely affected by the epidemic (30); for example, the economy slowed down, tens of thousands of people died, and government’s credibility declined. In addition to this, the pandemic exposed some shortcomings in the Chinese government’s response to public health emergencies. The Government of China promulgated the Civil Code during the pandemic, and many adjustments were made to the official Civil Code as compared to the Civil Code (Draft); these changes have a direct correlation with the COVID-19 pandemic. The pandemic profoundly affected the codification of China’s Civil Code, which also provided legitimacy to measures taken during the prevention and control of epidemics.

The COVID-19 provisions of the Civil Code draw on the lessons learned from the Chinese Government’s experience in epidemic prevention and control and are of great significance to the improvement of the country’s governance capacity. It is undeniable that there are still some flaws in the COVID-19 provisions of the Chinese Civil Code, such as the provision of temporary guardianship. In addition, the legislature has overemphasized the impact of the COVID-19 pandemic on the Civil Code, while neglecting the need to keep the legal provisions logical and streamlined. The study dialectically analyzes the innovations and deficiencies of the COVID-19 provisions in China’s Civil Code and provides reasonable modification suggestions for the deficiencies. Furthermore, we believe that it is not enough to emphasize only the adjustment of the Civil Code to public health emergencies and that it should be coordinated with the criminal law, administrative law, environmental law, and other legal norms to establish a perfect legal system for the emergency management of public health emergencies.

The world is experiencing a great change that has not been seen in 100 years, public health emergencies such as the pandemic will not be the last, and all kinds of security problems will bring new tests. The COVID-19 pandemic highlights governments’ limited capacity to govern in critical areas (31). The pandemic has no borders, countries are interconnected and interdependent, and all countries should unite and cooperate in their efforts to build a community of human health (32).

## Data Availability

The original contributions presented in the study are included in the article/supplementary material, further inquiries can be directed to the corresponding author.
